# Managing the Wellbeing of Elite Rugby Union Players from an Occupational Safety and Health Perspective

**DOI:** 10.3390/ijerph191912229

**Published:** 2022-09-27

**Authors:** Yanbing Chen, Conor Buggy, Seamus Kelly

**Affiliations:** 1 Michael Smurfit Graduate Business School, University College Dublin, A94 XF34 Dublin, Ireland; 2 School of Public Health, Physiotherapy and Sports Science, University College Dublin, D04 V1W8 Dublin, Ireland

**Keywords:** health and safety, injury prevention, risk acceptance, elite rugby union, player welfare, decision-making

## Abstract

The intense, physical contact nature of rugby union often encourages the normalization of risk-taking behaviour resulting in a relatively high acceptance of risk. This study aims to explore safety culture in rugby union from an OSH perspective, with the purpose of assisting coaches and management in their decision-making processes to improve players’ health, welfare, and long-term well-being. In terms of data collection, this study involved semi-structured interviews with senior support staff (*n* = 15) in elite rugby union. Interview transcripts underwent inductive analysis prior to an abductive analysis that was guided by an established occupational-safety-and-health (OSH) framework. Rugby union players’ safety can be considered from two dimensions: management’s commitment to safety (i.e., safety prioritization, safety empowerment, and safety justice), players’ involvement in safety (i.e., safety prioritization, and trust in other players’ safety competence, and players’ safety concern for the opposition players). Within the themes identified, players’ attitude towards their opponents’ safety which has been rarely considered as a factor for injury prevention is also discussed in this study. If sport support staff (i.e., managers/coaches/medical) can become more involved in players’ performance-orientated training using OSH management processes to aid in their decision-making, their exists the capacity to benefit players’ safe return to play after injury rehabilitation. Meanwhile, directing the development of appropriate behavioural educational interventions to raise safety-awareness amongst players can improve their long-term health and well-being and provide them with the necessary safety and health information to support their own decision-making processes. As a multidisciplinary design, this study contributes new multidisciplinary insights that have the potential to advance managerial practices utilizing an OSH perspective, including decision-making supporting risk alleviation for safety and long-term health and wellbeing initiatives in competitive team sports.

## 1. Introduction

Sport possesses unique cultural characteristics which quite often ‘revolve around extremely strong playing achievement and success orientations’ [1]. Rugby union is a popular sport worldwide and is considered a distinct workplace with its own culture, behaviours and norms that can have a substantial influence on players risk-taking decisions [2]. As a high-contact, physical, and often aggressive sport, players are exposed to an occupational risk of injury and illness [3]. In Ireland, prior to the pandemic, the overall match time-loss injury incidence rate for the men’s AIL (All-Ireland League) was 49.7/1000 player hours [4]. Specifically, the match time-loss injury incidence rate was 50.5/1000 player hours for men’s AIL Division One, and 48.7/1000 player hours for men’s AIL Division Two [4]. Moreover, rugby union players, like soccer players, often try and hide their injuries to remain in the sport [5] which emphasises that risk, pain, and injury should be accepted, tolerated, and played through [6,7]. Consequently, we aim to explore the situation by incorporating OSH into sports, initiating from Irish rugby union, which can potentially point the way forward for application in a wider range of sports internationally. Moreover, players often decide to prioritise performance over their health and safety, and it is not unusual for elite level rugby union players to take risks and display extreme competitiveness and aggression [8]. Moreover, players often underestimate how the consequences of these decisions may have serious short-term impacts and more long-term health and wellbeing impacts for their post-rugby union lives [9]. While recent debates reflect a clash of values on risk acceptability in sport and rugby union in particular [10], several aggregate factors influence rugby union players’ decisions to adopt risk-taking behaviour [4,8,9,10].

While young players’ participation in sports is inevitably influenced by their parents’ concern [11], elite players are concerned about professional, career and financial rewards [12]. Biomechanical risk factors can also be associated with injuries that limit athletes’ preparation and performance in certain type of sports [13]. In rugby union, however, due to the prevalence of injuries, chronic insecurity and vulnerability are prominent characteristics of players’ daily lives [14,15]. Consequently, players will often decide to cover up, or play with, an injury to advance their career and when faced with forthcoming contract negotiations with their employers. Players may also experience pressure from spectators and fellow teammates and conform to cultural expectations of being tough, physical and deciding to play regardless of pain or injury [16,17]. Professional rugby union is a results-based industry and coaches are the subject of considerable scrutiny from industry stakeholders such as club directors, media and fans concerning team selection, style of play and because a manager’s tenure is highly dependent on team performance and success they can often pressurize ‘key players’ to play while injured. Moreover, pressure from coaches regarding injury disclosure is widespread in sports [18] and sometimes when making team selection decisions coaches put medical staff under pressure to get an injured (key) player back to play before medical clearance [19].

While effective management programmes can facilitate enhancing positive health outcomes for players [20], limited research has focused on risk awareness and risk management in decision-making processes in professional team sports from an OSH perspective [5]. Reaching solutions based on an innovative understanding of complex situations in high performance sport, multidisciplinary research is deemed essential [21]. Rugby union-based health-promotion research has largely focussed on injuries, particularly issues that have garnered media attention, such as concussion awareness [22], technique-related injury-prevention [23], risk factors [24] and injury impacts [14]. However, relatively few studies have specifically examined rugby union [25] drawing on OSH risk awareness and management factors from an overall health-and-wellbeing perspective. Forms of risk management such as OSH management practices that have the potential to improve players’ health and long-term well-being are yet to be explored and are just beginning to attract academic attention [26]. For example, the management-player relationship has a crucial impact on a player’s decisions to report injury. Because players tend to avoid acknowledging the risks they face, health and safety-related communication between management and players is crucial and should be based on collaborative decision-making that involves balancing performance goals and players’ health conditions. To foster a culture that favours positive health outcomes for players, understanding how effective management programmes facilitate such positive outcomes is essential [20]. To achieve this, OSH risk-awareness perspectives suggest examining the types of behaviours and attitudes that are associated within an organisation’s safety culture [27]. In response, this study, through a consideration of risk-awareness and management perspectives sought to obtain useful and novel insights [28] regarding effective decision-making supporting the management of player health and welfare, taking elite Irish rugby union as an example. Moreover, insight into risk-awareness and management practices for sport support staff is fundamental to ensure the health-and-wellbeing of athletes as well as their support networks. 

This paper has two significant contributions. First, our theoretical starting point for this study begins by engaging with the key factors that are central to OSH practices relating to risk-awareness and management. No studies have specifically examined terms associated with health and safety-awareness (i.e., OSH risk-awareness and management factors) or the policies, procedures, and practices adopted by key rugby union support staff (i.e., medical staff, coaching staff, and other management personnel). Consequently, this study considers OSH risk-awareness and management principles in elite rugby union and provides a theoretical framework containing key OSH risk-awareness components customised to the context of rugby union. Utilising and applying OSH risk-awareness and management principles in sport is important because it can aid the explanation of why some players accept and adopt risky and aggressive behaviours. Second, theoretical health and safety-awareness components underpinning workplace activities are relevant and applicable by assisting practitioners in the creation of educational mechanisms and communication protocols that facilitate the development of OSH-focussed management principles in rugby union contexts. Exploring risk-awareness and management in sport (specifically in rugby union) from an OSH perspective facilitates an understanding of the conditions, processes, and consequences of making decisions surrounding risky behaviours in this unique workplace. This can then assist sport clubs, organisations, and their stakeholders in managing the potential detrimental and dysfunctional impacts that risky behaviours may have on employee welfare and development. 

Following a review of the theoretical framework guiding the analysis and operationalisation of the research, an overview of the methods is described. Then, the study’s findings are presented and discussed, followed by practical implications.

### Theoretical Framework

#### Safety Culture from OSH Perspective

Two interrelated core OSH concepts guide the analysis and operationalisation of this research: safety culture, and safety climate. Safety culture consists of shared values, attitudes, perceptions, and beliefs that drive decisions and behaviours regarding safety and can be manifested by human workplace behaviours in the organisation [29]. When a positive safety management culture is fostered in an organisation, positive behavioural change can be adopted by employees in sport organisations [5]. Safety climate can be seen as a reflection of safety culture in the organisation at a certain point of time [30]. Organisational safety culture is often viewed as the metaphorical personality of an organisation, whereas safety climate is often viewed as the organisation’s current mood [31].

Senior management’s commitment and leadership for employee wellbeing is a key driver of both safety culture and safety climate [32] and management should be willing to prioritise employees’ health and safety, rather than production or indeed performance [30]. Moreover, employee participation in safety-related decision-making and activities is a crucial indicator of an organisation’s health and safety culture [33]. Employees’ safety motivation and adoption of safety behaviours is largely determined by the safety standards and safety compliance activities promoted by leaders in their organisation [34]. In addition, safety empowerment is another important concept in this domain; it refers to delegating power to allow employees to have flexibility and meaningful input in decision-making regarding health and safety [35]. Safety empowerment accords with RTP (Return to Play) and if management do not give players opportunities to make safety-related decisions, players may hide injuries rather than proactively report them. In rugby union, safety empowerment demonstrates that management trust players’ safety-related judgment, and can be applied from decision-making, through play, to the treatment of minor injuries. A primary constituent of workplace safety is co-operation through empowerment, mutual trust, and two-way communication, in which good management-employee relationships are essential, both personally and professionally [36]. The problem in some sport organisations is that risk-acceptance often plays a negative role in safety prioritisation, counteracting active safety behaviours [37], and the prioritisation of safety often counteracts aspects of player performance.

Several studies have explored aspects of culture in elite sport teams and identified that the culture of many sports encourages players to adopt aggressive behaviours [38], to compete regardless of pain or injury [39], and to tolerate pain and injury in order to maintain a disciplined body or athletic image [40]. Moreover, the ‘normalisation’ and ‘rationalisation’ of risk, pain, and injury [41] have been invoked as part of the ‘sport ethic’ [42], or ‘culture of risk’ [6], even in non-contact sport contexts [43]. However, few studies have explored the importance of safety culture in a team sport or sport organisation from an OSH perspective. With the purpose of exploring health and safety awareness in competitive team sports, this study contributes new insights that have the potential to evolve and advance managerial practices including health related decision-making to support risk alleviation from an OSH perspective. As discussed, players and stakeholders (e.g., coaches, teammates, family, and friends) in their ‘sportsnets’ or sports-related social networks [6] often encourage and endorse values and beliefs associated with a culture of risk [44,45,46]. This culture of risk emphasises that risk, pain, and injury should be accepted, tolerated, and played through [6,7]. This culture of risk could be considered the antithesis of how OSH management evaluates and negates risk in order to ensure that employees do not have to accept or tolerate injury or ill-health. Performance is often prioritised relative to player safety, which can also be reflected by the ‘relative lack of influence’ of sports medicine [16]. In previous research, some coaches were found to dispute or negotiate medical recommendations [45,47]. Medical staff in ‘sportsnets’ often have relatively limited power over their athlete-patients [48], as they are required to balance ethical medical considerations with their employers and/or coaches’ demands [49]. Considerations of ‘precaution’ and ‘risk’ [44,47], and of health-and-wellbeing in terms of performance [50] are not always prioritised. In recent years, the important role of sport medicine has been gradually acknowledged due to issues surrounding concussion [51,52]. In such a circumstance, the existing framework for clinicians to improve the decision-making process in return to sport [53] may not work efficiently due to the power imbalance between key stakeholders in rugby union.

## 2. Materials and Methods

This study is part of a larger published research project using a mixed-methods approach, beginning with a participant observation on rugby training sessions and competitions that was incorporated as an ethnographic study [12], and followed by a quantitative research survey developed for collecting data directly from rugby players [2]. Qualitative research is about examining people’s lives in rich detail [54,55]. Consequently, in this study, semi-structured interviews with key stakeholders were considered the most appropriate method for the gathering of rich, in-depth information and exploring participants understanding and interpretation of the specific research questions being explored [56]. This study was grounded in a critical realism (CR) approach [57]. This approach aligns with CR’s ontological realism, meaning, reality exists independent of our knowledge of it, and epistemological constructivism, which argues that the means through which we acquire the truth are limited [58].

### 2.1. Participants

In line with recommendations [56] the aim was to recruit a sample of participants with diverse perspectives, backgrounds, and experiences. Participants in this study were employed in high-performance sport teams with elite sport athletes at the time of interview. Participants were in line with similar elite-focussed studies in Irish rugby union [59]. Specifically, elite rugby union teams in Ireland comprise national, provincial (i.e., Pro14), and AIL 1A teams (i.e., All-Ireland League Division 1A). Utilising a snowball-sampling method [60], 15 participants were recruited from a purposive sample of support and management in elite rugby union teams. At the time of the interviews, all participants were full-time (*n* = 10) or part-time (*n* = 5) support staff in elite Irish rugby union; categorised as medical staff (*n* = 5), coaching staff (*n* = 6), and/or other management staff (*n* = 4). Additionally, they represented three levels of the sport (national [*n* = 7], provincial [*n* = 3], and AIL 1A [*n* = 5]). Demographic information is presented in Table 1. Determining the “correct size” of a qualitative sample can be a problematic issue in qualitative research [61]. Rather than breadth, our aim was to recruit participants based on relevance and variations in the participants in terms of their demographics, characteristics, and experiences [61]. Consequently, participants were selected that could provide more relevant data and a depended understanding based on their ‘connection to’ and ‘involvement in’ the research topic [61]. We argue that the sample size provides reasonable coverage and rich, in-depth data was collected from information-rich participants [61] leading to a greater level of understanding of the research aims. The total number of participants was deemed appropriate based on attaining data and meaning saturation [62] and following consultation with the expert research studies panel that acted as peers for the design and development of this research project. We acknowledge that non-random samples may lack representativeness, are non-probabilistic, lack statistical power and may lack generalisability [61]. However, the sample limitations may be offset by the highly specialised nature of the participants and their knowledge and experience with the current research topic. 

### 2.2. Study Chronology

#### 2.2.1. Data Collection

Following a review of OSH and sport-related literature, key interview topics concerning OSH issues in sports were initially identified [5]. In relation to the array of safety culture frameworks reviewed [64], a well-established and leading quantitative research instrument within the field of OSH climate/culture evaluation, namely the NOSACQ-50 safety-climate questionnaire [37], was identified with the potential to be applied in sports. Based on its solid theoretical framework, the NOSACQ-50 has been widely applied in various occupational settings internationally and validated in over 40 languages. The key factors that influence safety culture considered in the NOSACQ-50 framework were utilised to inform the development of the interview guide. The interview guide (Appendix A) comprised of questions regarding health and safety-related awareness, perceptions, practices and was piloted prior to data collection. The first author conducted all the interviews, which were audio-recorded with participants’ written consent, and in accordance with the ethical exemption granted by the human research ethics committee of the study team’s affiliated university. Logistical issues regarding suitable times and places for meetings resulted in one interview being conducted through online communication (i.e., Skype). 

#### 2.2.2. Data Analysis

The first author transcribed all interview content verbatim and sent the transcripts to the participants for review and accuracy verification [65]. Four participants provided additional feedback, which was used to update the relevant transcriptions prior to data anonymisation. 

The initial analysis was data-driven, comprising inductive thematic analysis [66]. Firstly, the first author familiarised herself with the transcripts by repeatedly reading them while also writing reflective notes and tagging meaning units using software NVivo 11. Subsequently, she generated codes by implementing an iterative process; codes were initially clustered into lower-order themes, and then into higher-order themes to represent the data. After a satisfactory thematic map was developed, the abduction process commenced.

**Figure 1:**
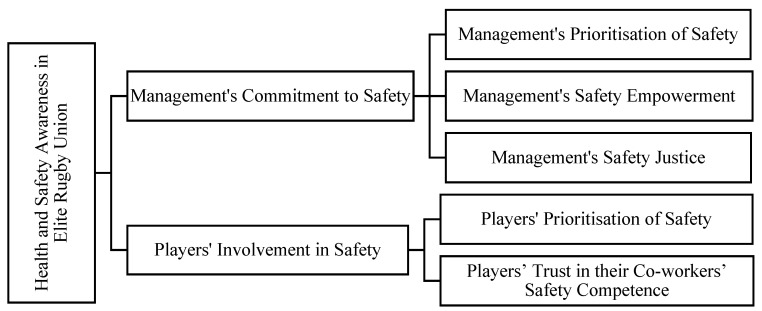
Health-and-Safety-Awareness Framework in Elite Rugby Union.

In the abduction process, key themes were conceptualised by incorporating the NOSACQ-50 framework with necessary modifications to customise to the rugby union setting (Figure 1) at two levels: higher-order themes to organise and structure the analysis and lower-order themes to report the meaning of central concepts. Finally, lower-order themes were represented with quotes from interviewees to illustrate the findings. 

### 2.3. Trustworthiness

Credibility and rigour were pursued throughout this study, based on the principles of CR in qualitative research [58]. Firstly, all participants were senior support staff with considerable knowledge and experience of the phenomenon under investigation. Through the snowball-sampling strategy, participants were screened to ensure that a variety of roles in elite rugby union and multiple levels were represented. Secondly, all interviews were conducted by one researcher who consistently used the same interview guide to minimise any potential discrepancies [67]. Thirdly, an abductive analysis approach was applied during data analysis, beginning with an inductive process that avoided a priori sports theory interference, and completed using a deductive process that enabled key findings to be systematically conceptualised [68]. All co-authors acted as ‘critical friends’ by challenging the data coding, categorisation process and in reviewing the identified themes [62]. Finally, substantial descriptions on the themes identified were provided to allow readers to judge the potential transferability of the findings. According to the CR approach, different levels of truth was considered throughout the analysis, as the data obtained from the participants are inevitably linked to their respective experience.

## 3. Results

As the present research was based on a safety-climate framework, findings are presented from the dimensions of rugby union management’s commitment to safety and rugby union players’ safety involvement.

### 3.1. Management’s Commitment to Safety

#### 3.1.1. Management’s Prioritization of Safety

The theme ‘prioritisation of health and safety by management’ in rugby union can be manifested through risk-prevention activities and risk-impact control measures after accidents. In rugby union specifically, it can be reflected through key safety-related practices, such as safety techniques arranged in daily training routines and attitudes towards RTP. One of the medical staff gave examples of management’s efforts to minimise risk:

*“It’s monitored, say somebody’s appetite is always 10 out of 10, and suddenly drops to six; an alert will pop up. If their sit-and-reach is normally 10 centimetres and suddenly drops to five, an alert pops up and physios are called straight over. Maybe we’ll look at them [the player] to try to pre-empt injury.”*

(Med12)

The ‘it’ Med12 refers to at the beginning of the quote represents health-related behavioural data that are monitored via an online app. Besides organising and monitoring players’ physical exercise, mental preparation, and lifestyle, managers also communicate with medical staff to prepare for possible injury situations [69]. Medical staff also discuss with team management on players’ health and safety regarding the provision of injury treatment during a game. As Mgt1 mentioned:

*“At the start of the season, our doctors and physios hold a meeting to decide on the procedures that will be implemented in different situations, then communicated between coaches”*.

However, managers face difficulties upholding ‘duty of care’ when players’ safety conflicts with performance expectations regarding workloads. The Irish Rugby Union Football Union (IRFU) implemented control mechanisms to monitor players’ workloads during competitions [70], which may not be available in other countries as Med12 stated:

*“IRFU try to manage somebody across the whole season, and say nobody can play every game. ‘How many games can we get this person to play, so they are healthy at the end of it?’ I think that’s something very important that other countries and unions don’t necessarily do”*.

Although safety communication should constitute a reciprocal relationship between management and players. However, management often need to initiate this communication since players may not acknowledge the risks they face. For instance, Med4 discussed the communication with players on injury-reporting:

*“Then, after games, I go chat to the players who played, see if anyone’s picked up any knocks [injuries] that they didn’t mention, during the game”*.

Whether a player can participate in a game largely depends on the prognosis (or diagnosis) of medical staff, especially when the player has not fully recovered from an injury. However, post-injury RTP is usually based on collaborative decision-making and involves balancing performance goals and players’ health conditions. However, as Med3 described: ‘That’s kind of a grey area, might be too idealistic to say that it always rests with the medical team, because the medical team’s activity is going to be pretty conservative’. As performance becomes increasingly competitive, players’ safety can be compromised to increase the possibility of winning a game; thus, conflicting opinions between medical and coaching staff are common:

*“Obviously, the pressure of sports probably gets rugby coaches to put the medical staff under a bit of pressure, especially with the one-of-a-kind main player that they need in the squad on a certain weekend. But I think that’s probably natural, in this sport.”*

(Coach6)

Sometimes, coaches put medical staff under pressure regarding post-injury RTP [16]. The desire of the coach to succeed can be a source of pressure for a doctor to get an injured player back to play before medical clearance [19]. Interestingly, this phenomenon was revealed by Coach6 as a common behaviour, which should have been reported by medical staff as an unethical behaviour. In contrast, the medical staff participating in this study contributed numerous examples of how player safety was prioritised. For example, several medical staff participants identified the importance of monitoring health-related data (Med12), regulating and controlling player workloads (Med12), performing on-pitch worst-case scenario analysis (Med3), and post-match injury follow-up (Med4). Moreover, Med3 accepted the ‘fact’ that medical prognosis is usually conservative in rugby union. Underpinned by CR that the truth acquired is limited and considering the possibility of the relative ‘lack of influence’ of sports medical staff in decision-making indicated by previous research [16], medical staff may not have reported everything they knew during the interview.

With the increase in competitiveness and commercialisation marked by the rising importance of winning, sport medicine has been considered as a way of optimising performance rather than health-and-wellbeing. From an OSH perspective, there is an urgent need for sports such as rugby union to foster a culture that enables support staff to openly discuss safety-related concerns to aid in decision-making without pressure. 

#### 3.1.2. Management’s Safety Empowerment

Safety empowerment demonstrates that management give the players autonomy and involve players in making safety-related decisions. As Med5 indicated:

*“If I substituted every player with any sign of injury, there would be a lot of changes during a game. But, I’m giving them opportunity to prove they’re okay to continue to play. And they respect that, when it comes to making big decisions”*.

This quote from Med5 reflects a previous finding that, in the construction industry, a primary constituent of workplace safety is co-operation through empowerment, mutual trust, and two-way dialogue [36]. Safety empowerment accords with RTP. In other words, if management does not give players opportunities to make safety-related decisions, players may hide injuries rather than proactively report them. Thus, the management-player relationship has a crucial impact on players’ injury-reporting behaviour. If players are highly aware of their health and safety, management can more easily obtain accurate health-related information from them. However, as players may not always prioritise their safety, to ascertain players’ health conditions, management must build personal relationships. As Coach8 explained: ‘It’s like building that relationship with people. There’s a kind of professional side. There’s a time to laugh and joke with them after a match… because you don’t want to just be a dictator.’ Similarly, Med13 reported:

*“We try and maintain that relationship, and have professional interactions with them so that they see that we are professionals, not just some friends, we are trained professional people giving you what we believe is good advice”*.

The coach-player relationship resembles a balancing act, as coaches must maintain professionalism, but also be encouraging and helpful. Conversely, players may under-report injuries, as they may fear that reporting an injury may imply a lack of fortitude or will prevent them from playing, both of which may negatively impact their career prospects:

*“It’s a tricky area. Obviously, player-to-coach is a critical relationship in terms of building a team. It’s different because, you’re thinking, ‘if I say this to the coach, will he think that I’m soft?’ Or ‘will he think that I’m weak?’ Or, ‘will he think that this is a real injury or that I’m looking for an easy way out?’”*

(Coach11)

Coach11 had elite playing experience, and he therefore acknowledged that players may withhold injury concerns if coaches did not give them sufficient trust and recognition. The empowerment topic has been frequently discussed in coach-player relationships regarding performance. For example, an athlete-centred coaching style that encourages players to take greater ownership and responsibility of their behaviours has a beneficial impact on players’ wellbeing and performance, which has been valued by New Zealand Rugby [71]. In parallel with OSH, the empowerment of employees to discuss safety concerns or ideas through an open and receptive leadership is also a positive safety culture hallmark.

#### 3.1.3. Management’s Safety Justice

Rugby union referees penalise players who contravene the game’s safety rules which represents safety justice. If a player is injured, the referee should identify this quickly and allow medical resources to provide timely treatment; players who have good safety-awareness will not hide injuries and mislead the referee.

*“From a referee’s perspective, one of our primary concerns is the health, safety, and wellbeing of a player. If something happens within the game, the correct reaction must be taken; even stopping the game in certain circumstances. If a referee doesn’t spot in advance, if an act of foul play is committed, depending on the severity of the act of foul play, the referee should react in an appropriate way by either sanctioning a yellow card or sanctioning a red card. And although that doesn’t stop the event taking place, we can prevent further events taking place.”*

(Mgt10)

Intentional fouling is understandable, considering the huge motivation to win, but this is often an overlooked risk factor for sports-related injury [72], as Coach14 stated: “*For example, if a referee isn’t [penalising/judging] a hard tackle appropriately, that might mean that more hard tackles will be allowed… which could result in an injury*”. In professional English Rugby Union, only 6% of illegal/dangerous tackles were correctly penalised in accordance with the game rules [73]. Thus, players can intentionally hurt opposition players without the referee deeming this foul play; this especially occurs when players become familiar with a referee’s arbitrary style: “*Obviously, there are different styles of referees. Some are very strict on dangerous play, and other referees can be… a little bit lenient*” as Mgt1 explained. 

The participants felt that assessing a referee’s performance and decision-making style is an indispensable part of competition tactics. In particular, one participant identified that top-level rugby union teams often devote considerable resources to studying referees’ performances. Mgt7 gave the examples: “*Another level is to analyse the referee’s performance. What’s his strength? What does he look for? And what does he emphasise?*” Coaching staff may also interfere with a referee’s decision-making to increase their team’s chances of winning a game and many match situations represent challenges for referees regarding correctly applying the rules of the game. Consequently, rules are continuously being revised and changed:

*“Refereeing in rugby is hugely challenging, be it amateur or professional. It’s a hugely intelligent game, because the laws, in some instances… are quite black-and-white, but in other instances, grey. The challenge for referees is to find the black-and-white in… a grey situation.”*

(Mgt10)

A case of unintentional high tackle during a Heineken Champions Cup game was mentioned by Coach15 as an example:

*“The perfect case is Danny Cipriani, playing for Gloucester against Munster two months ago. So there are a lot of decisions that the referees are trying to make… but the changes are hard to make”*.

The complex nature of the game means that rugby union referees have an increasingly wide range of responsibilities regarding players’ injury risk. Considering the dilemmas referees may be confronted with, it is crucial for players to improve their health and safety-awareness and engage in safe practices. 

Traditionally, referees are rarely considered as an important factor in managing player safety in sports. However, from an OSH perspective, a referee can make safety judgements in these grey areas on the field in the heat of the moment and take on a leadership role in ensuring safety justice for players.

### 3.2. Players’ Involvement in Safety

Rugby union players’ participation in safety-related decision-making activities and involvement in safety practices were identified. Specifically, two themes were identified: players’ prioritisation of safety and players’ trust in their co-workers’ safety competence. 

#### 3.2.1. Players’ Prioritisation of Safety

Risk-acceptance usually plays a negative role in safety prioritisation, counteracting active safety behaviours [37], and prioritisation of safety often counteracts aspects of player performance. A typical situation is RTP decision-making regarding an injury. Injury can hinder players’ performance and impact their wellbeing, as Coach11 described:

*“Injury has a bigger impact than just missing a couple of training days. You’ve got the mental side of it too. Because it troubles you, makes you think… if you’re going to feel down, you’re not going to be happy about things, and it’ll affect you in other ways.”*

(Coach11)

In rugby union, full recovery is not always deemed necessary before RTP, nor are protocols and procedures always followed [74]. Despite efforts to develop a unified definition of a rugby union injury [75], the boundary between major and minor injuries often remains unclear. The decision to play with an injury is subjective, as Coach 14 explained,

*“Someone has a niggling injury… may not be comfortable playing with it. Others maybe have a higher pain threshold or are prepared to train with a little bit of discomfort”*.

Players’ health and safety may not be prioritised if they have a strong motivation to play. As Med4 reported: “I know that there’s some players who talk to each other if they have knocks, but they might not tell the coach, or the doctor, or the physio because they might think if… someone thinks they’re injured”. Med4 mentioned that rewards from playing are key motivations. To counter injury pain while playing, players may use painkillers, as Coach6 noted: “If it’s a certain type of injury, you’re able to play without really doing too much damage, you can get an injection in your shoulder to mask the pain and get through another game”. Players may even risk playing with serious injuries such as concussion, as Med12 mentioned: “For concussion, we’ve done a huge amount of education on this, but they’ll tell you they’re fine because they want to play. We’re not just taking their word for it, they always tell you they’re fine”.

Med5 emphasised the importance of establishing mutual trust between medical staff and players, as such a relationship could foster timely injury treatment that can dramatically reduce the rate of unreported injuries:

*“You develop mutual trust… when you know players, they are honest with you and tell you how it is. When you don’t have that relationship with players, with health and safety, it’s very difficult to figure out whether that person is okay”*.

Similarly, Mgt7 discussed the stakes for coaches regarding their relationships with players: “*The coach’s performance is on the success of the team. In the professional era, if a team is continuously losing… the coach could be sacked*”. According to Coach14, decisions regarding whether a player can play with an injury should involve all parties:

*“instead of relying solely on players and physiotherapists to ensure health and safety-awareness, all staff in the organisation should contribute to decisions regarding players”*.

As safety culture is a part of organisational culture, many safety programmes in rugby union use the motto ‘safe technique is effective technique’ without realising that such mottos are a form of safety communication central to fostering a positive safety culture [23]. This indicates that the rugby union safety culture encourages players to develop an awareness of safety practices only insofar as they benefit the performance of the team:

*“I think part of that is the culture of the club. Outcomes can take a second place to player welfare for us. I think some of the barriers to implementing health and safety or welfare, I suppose the pressure on outcomes might be greater, there’s a player-welfare-focussed or performance-focussed judgement. If there’s pressure to perform, player welfare can be put to one side or pushed away.”*

(Mgt2)

In addition to pressure from coaches, players may also experience pressure from their teammates because

*“they don’t want to be seen to be the weak one. They want to be seen to be as good or as healthy as the next guy.”*

(Mgt7)

Pressure can also come from non-rugby union-playing peers, as Coach6 explained:

*“I think it’s just the pressure, like the pressure to keep your position and contract. You’re publicly exposed, it’s a competitive situation. People out there, they might have been in the same year in college, and they’re becoming qualified accountants, and they’re starting to make some money, but you’re not.”*

(Coach6)

Another theme concerned players’ motivation to obtain material rewards from playing, such as remuneration for appearances or prizes for winning games:

*“I think it’s because it’s sports, and your decision to play is by your contract, might actually be if you’re getting closer to contract negotiations, and you know you can play through injury, and cover up an injury as much as possible.”*

(Coach6)

However, in contrast, Mgt7 suggested that players’ motivation to play rugby union aggressively was based on financial reward and because rugby union players have short careers, they try to accumulate enough money to secure their futures:

*“The more competitions you win, the bigger the cash bonus or wage bonus is. Now, a lot of those players will say they love playing the game, and they’re not really worried about the cash. That’s not true, because their career is only 15 years at most, it’s probably less. They only have that period of time to amass as much income as they can. Earnings would be in the region of €300,000. Basically, that’s a lot of money for a 22- or 23-year-old player.”*

(Mgt7)

Mgt7 also mentioned the pressure on players concerning obtaining a new contract. Several participants mentioned that players tend to avoid missing games because of potential negative impacts on their career continuity, as Coach11 described*:*

*“A player might have a career that’s 10, 15 years, whereas I might have a career in an office job for 40 or 50 years. So, missing time through ill-health is much more significant for players”*.

Consequently, the culture within rugby union has a substantial influence on whether players prioritise performance or their health and safety, which may have long-term health and well-being impacts for their post-rugby union lives.

#### 3.2.2. Players’ Trust in Their Co-Workers’ Safety Competence

Players’ trust in their co-workers’ safety competence, including playing techniques, is important because tackling is the main cause of safety issues. Tackling technique was mentioned by most participants when discussing health and safety-awareness:

*“I suppose, if you take contact as a major issue, I think tackle technique is important. If you see someone tackling with their head across, and the person is hit… that can be a disaster. And you may see a lot of shoulder injuries by someone who is actually tackling passively.”*

(Med5)

Techniques such as tackling, mauling, and engaging in the scrum and lineout are part of the game, but also affect teammates’ health and safety. Players’ trust in their teammates’ safety ability is important, especially when players have differing training backgrounds. For example, Coach8 reported:

*“It’s quite unique in a situation when those players come in, because they may be playing, passing the ball, to someone who’s never played rugby like that before. So you’ve got the elite of the elite, and… amateurs play with them”*.

Trust in co-workers’ safety competence is a key indicator of safe performance. In general occupations, employees in the same workplace are co-workers, responsible for each other’s health and safety when working together. Rugby union teammates are supposed to care about each other’s health and safety as co-workers. Further, both sides in a match are also ‘co-workers’ because neither team can work (play) without an opposing team in the workplace (pitch). 

Players from both sides have a ‘duty of care’ to opposing players, thus ‘co-worker’ should include players on both teams, and players should be able to trust their co-workers’ safety competence. Despite this, players may find it difficult to compromise their aspiration to win a game to protect an opponent. Coach15 explained that intimidating the opposing team through aggression is a strategy for winning: “I don’t think anybody would ever want to injure another player… but you would always want to hurt them… if there’s a difference. So, you want to hurt the opposition and make them fearful and less committed to contact”.

Coach15 and Mgt7 believed that players’ personal relationships determine whether opponents should be treated equally to teammates in terms of health and safety. In Coach15′s view, the more familiar a team is with the opposing team, the more aggressive they may be:

*“Players, who know each other will often be more brave, be very aggressive with someone. But, if they don’t know the other person that well on a foreign team or another club, they might be a little bit reserved and sometimes coaches will have a small part of a training session during the week of a big game. They will say, ‘we’re going to go full metal jacket’. And that means that there are very few rules and people just get barbaric.”*

(Coach15)

In contrast, Mgt7 voiced an opposite opinion and suggested that players may not be as aggressive if their opponents are occasionally their teammates.

*“Every week you have rugby players play for Ireland, but next week in the league they’re playing on opposite teams, so they play against each other all the time. So, it’s difficult to be overly aggressive with your team, with your Ireland teammates. Your teammate is there, but the following week [he] could be your opposition, the opposition player.”*

(Mgt7)

However, teammates could also be treated as opponents during a player-selection training session. In this case, individual career aspiration is prioritised:

*“I don’t think any of those players would want to injure another player. But I have seen in Irish training sessions where one group of forwards will go against another group of forwards, and some of the players will almost try to injure their own teammates because they might get selected.”*

(Coach15)

As the interviewees repeatedly mentioned, even if no player aims to intentionally injure opponents, opponents’ (and even teammates’) safety is sometimes neglected when compared with the team goal of winning, or the individual goal of improving a sports career. From this perspective, players’ acknowledgment of their responsibility for the safety of their co-workers is vitally important for improving players’ health and safety-awareness. Practically, whether opponents are co-workers from a player’s perspective still requires further exploration. 

## 4. Managerial Implications

Because the culture of playing rugby union often conflicts with safety culture, it could be unrealistic to totally prioritise players’ health and safety over their performance. Specifically, as a management dimension, the overall culture in team sports that prioritises player performance rather than safety is often dominated by stakeholders represented by coaching staff, as previous research has indicated [16]. In comparison, medical staff, with an ethical commitment to player safety in sport teams, often have less power on related decisions. From a player’s perspective, individual recognition of safety can be limited by the prevailing culture in the sport team that encourages win-at-all-cost attitudes, with the individual player’s safety decision-making being impacted by the surrounding referents in the ‘sportnets’. Based on accepting these facts, managerial implications from an OSH perspective can be considered from the following angles. 

Firstly, like non-sport industries in which the outcome of prioritising safety can improve productivity and performance, medical staff can emphasise that player health and safety is the premise for achieving better performance. Aligned with an OSH management principle that risk should be controlled as low as reasonably practicable, there is supporting evidence that most intervention programmes incorporating safe techniques are effective [76], particularly in tackling and scrummaging [77]. If medical staff can become more involved in rehabilitation training, strength-and-conditioning, and even playing techniques, they can gradually increase their organisational leadership potential by providing professional medical expertise with the purpose of maximising performance. Their close work with coaching staff can thus potentially alleviate the marginalised position of medical staff in some sport teams. 

Secondly, since team sports players’ recognition of safety can be largely impacted by their surrounding referents, educational efforts focused on risk-taking, injuries, and long-term health should be initiated from individuals in management positions at the team, club, and national association levels. For example, World Rugby may mitigate risks by implementing game rule changes, and the IRFU have educated its players and coaches concerning concussion. At the club level, coaches and managers need to consider their communication mechanisms with players in relation to what constitutes aggressive behaviour, unnecessary risk, and reckless play, and why they are particular risk factors for some individuals and their teams. At a structural level, the role of rule changes concerning the competitive nature of rugby union has the potential to reduce risky play, especially regarding unsanctioned behaviours employed to intentionally injure opposition players [8], which will represent a move toward a safety culture within rugby union where respect and cooperation with co-workers is key. However, the contact nature of rugby union still supports and legitimises the competitive, physical, and often violent aspect of the game. Therefore, rugby union organisations should formulate safety-related campaigns customised to optimally promote safety-awareness for specific risks. Such campaigns can be tailored to educate both players and support staff on key risk factors of injury and long-term health-and-wellbeing; such communication practices would be a positive step towards ensuring that decision-making processes in these areas can contribute to the establishment a safety culture within rugby union. At a club level, safety practices can be initiated from coaching-based skills, including the knowledge and experience provided by strength-and-conditioning coaches and experienced players. This is effective to engage the players as their enthusiasm for performance skills would provide safety practice with an equivalent emphasis, while those closely interacting social referents can directly influence players’ safety-awareness. This multi-level approach to education incorporating clubs, national associations, and unions [78] can cultivate a safety culture in team sports. 

Thirdly, as previously mentioned, the role of the referee regarding on pitch in the moment decision-making processes, which may have been traditionally underestimated in maintaining safety justice, provides direct feedback on players’ on-pitch behaviour regarding their aggressiveness level. Their potential to be involved in the leadership of risk-awareness and management is apparent and should be explored in more detail in future research. Furthermore, OSH provides a distinctive insight that team sports can be safer if all opposition team players can be considered as ‘co-workers’. The trust in co-workers’ safety competence can also lead to safe playing technique, as poor techniques may injure the opposition players. However, even though a ‘duty of care’ has been incorporated in the rules of the game, it could be emotionally difficult for players to trust the opponents as ‘co-workers’ regarding safety play during a competitive game. Instead of imposing the concept that the opponents should be treated as ‘co-workers’, the concept of respecting and trusting opponents to consider risk can be a mechanism to build an inter-organisational safety culture across the sport. Further research is required to explore players’ safety concern for their opponents as a form of safety intervention. 

## 5. Conclusions

This study provides new insights into aspects of player welfare in elite rugby union through the consideration of an OSH-based theoretical framework that can assist in informing and framing the decision-making processes adopted by elite team managers, coaches, and medical personnel. While the findings are specific to these participants and cannot provide a basis for generalisations to other populations (i.e., other elite team sports) there is the possibility of naturalistic generalisations [54] to other national cultures where rugby union is played, and to similar sports, thus allowing a platform for further comparisons. Similarly, attitudes towards rugby union-performance rewards may vary depending on social welfare, post-career opportunities, overall sports income/welfare, etc. There is a growing trend towards uniformity in the development of elite sports in western countries, but a certain diversity remains in individual countries [79], particularly in East Asia. While this study was restricted to a single nation’s rugby union context, Ireland has one of the oldest and best-established rugby union organisations and team structures globally, alongside a robust legal and practical OSH regulatory framework enables Ireland as an example for rugby union organisations globally. However, to fully ascertain whether this study’s research framework can be applied in emerging rugby union contexts, samples are required through future research from countries such as China, where health and safety management is neglected in most occupational contexts and has virtually no influence in elite sport [80]. 

Given that the five themes identified (Figure 1) in this study were interrelated, more detailed statistical procedures, such as factor analysis or principal component analysis, could be implemented in future quantitative research. For example, a management team’s safety priority may generally align with players’ individual safety priorities, and be influenced by player performance, which could emerge as a new dimension linking performance with safety. Additionally, as rugby union is a team sport, future research should note that individual players’ opinions or understanding of their own health and safety (and the decisions they make in that regard) as well as that of their teammates (and potentially opposition players) can be influenced by their teams’ cultural response to safety. Team culture is usually developed through relationships between players and management/support staff; therefore, associated factors may include team attitude towards injury (e.g., preventable/normal) and risk behaviour (e.g., honourable/dishonourable); willingness to prioritise safety over winning; trust in team players’ playing/safety techniques; and availability of medical services and their input to safety management. A referee’s function in establishing a positive safety culture in rugby union also requires further exploration. In terms of the cultural dimension, because rugby is a high-contact and often aggressive sport, a safety culture and safety climate need to be implemented by senior management. This is important because the organizational dimension that puts pressure on players to perform often encourages the normalisation of risk-taking behaviour by players resulting in the prioritization of performance over their own safety. The adoption of a safety culture and safety climate can assist key stakeholders in managing the potential detrimental impacts that risky behaviours may have on employee welfare and development.

These study findings support the creation of educational mechanisms and communication protocols that facilitate the development of OSH-focussed management principles to support decision-making regarding player health, wellbeing and safety in rugby union. This study also supports the development of surveys and/or interventions for determining the status of health and safety perception and awareness among elite rugby union players and raising risk-awareness which can inform their own individual decision-making. This will facilitate the development and introduction of long-term health-and-wellbeing interventions. It is hoped that this research contributes, in part, to existing academic discussions concerning the dynamic nature of athlete welfare and safety.

## Figures and Tables

**Table 1:** Participants’ Background Information.

Participant Pseudonym	Position Involved	Team Level (Highest)	Employment Type	Age Group	Gender	Elite Playing Experience
Mgt1	Management	Provincial	Full-time	50–60	M	Yes
Mgt2	Management	Provincial	Full-time	30–40	M	Yes
Med3	Medical staff	National	Full-time	30–40	M	No
Med4	Medical staff	AIL 1A	Part-time	20–30	M	Yes
Med5	Medical staff	AIL 1A	Part-time	30–40	M	No
Coach6	Coaching staff	Provincial	Part-time	20–30	M	Yes
Mgt7	Management	AIL 1A	Full-time	50–60	M	Yes
Coach8	Coaching staff	AIL 1A	Part-time	20–30	M	Yes
Coach9	Coaching staff	AIL 1A	Part-time	40–50	M	Yes
Mgt10	Management	National	Full-time	40–50	M	No
Coach11	Coaching staff	National	Full-time	20–30	M	Yes
Med12	Medical staff	National	Full-time	30–40	F	No
Med13	Medical staff	National	Full-time	50–60	M	No
Coach14	Coaching staff	National	Full-time	30–40	M	Yes
Coach15	Coaching staff	National	Full-time	40–50	M	Yes

Notes: 1. Management include referee, team manager, director, coordinator, etc. 2. Medical staff include team doctor, physiotherapist, medical consultant, medical coordinator, etc. 3. Coaching staff include head coach, strength and conditioning coach, assistant coach, coach’s coach, performance analyst, etc. 4. AIL 1A = All-Ireland League Division 1A. 5. This table was cited from our previous publication [63].

## Data Availability

Not applicable.
